# Bi-weekly eribulin therapy for metastatic breast cancer: a multicenter phase II prospective study (JUST-STUDY)

**DOI:** 10.1007/s12282-018-0843-y

**Published:** 2018-02-12

**Authors:** Shoichiro Ohtani, Takahiro Nakayama, Tetsuhiro Yoshinami, Ken-ichi Watanabe, Fumikata Hara, Yasuaki Sagara, Hidetoshi Kawaguchi, Kenji Higaki, Nobuki Matsunami, Yoshie Hasegawa, Masato Takahashi, Makiko Mizutani, Takashi Morimoto, Masako Sato, Mitsuya Itoh, Satoshi Morita, Norikazu Masuda

**Affiliations:** 1 Division of Breast Surgery, Hiroshima City Hiroshima Citizens Hospital, 7-33 Motomachi, Naka-ku, Hiroshima-shi, Hiroshima 730-8518 Japan; 2 Osaka Medical Center for Cancer and Cardiovascular Diseases, 1-3-3 Nakamichi, Higashinari-ku, Osaka-shi, Osaka, Japan; 3 NHO Hokkaido Cancer Center, 3-54 4-jo 2-chome Kikusui Shiroishi-ku, Sapporo-shi, Hokkaido Japan; 4 Department of Breast Oncology, NHO Shikoku Cancer Center, 160 Kou, Minamiumemoto-machi, Matsuyama-shi, Ehime Japan; 5 Division of Breast Surgery, Hakuaikai Medical Corp. Sagara Hospital, 3-28 Matsubara-cho, Kagoshima-shi, Japan; 6 Matsuyama Red Cross Hospital, 1 Bunkyo-cho, Matsuyama-shi, Ehime Japan; 7 Higaki Breast Clinic, 8-23 Hondori, Naka-ku, Hiroshima-shi, Hiroshima Japan; 8 Osaka Rosai Hospital, 1179-3 Nagasonecho, Kita-ku, Sakai-shi, Osaka, Japan; 9 Department of Breast Surgery, Hirosaki Municipal Hospital, 3-8-1 Omachi, Hirosaki-shi, Aomori Japan; 10 Department of Surgery, Breast Oncology, NHO Osaka National Hospital, Chu-ouku Hoenzaka 2-1-14, Osaka-shi, Osaka, Japan; 11 Yao Municipal Hospital, 1-3-1 Ryuge-cho, Yao-shi, Osaka, Japan; 12 Kyoto University Graduate School of Medicine, 54 Kawahara-cho, Shogoin, Sakyo-ku, Kyoto, Japan

**Keywords:** Eribulin, Schedule modification, Metastatic breast cancer, Bi-weekly schedule

## Abstract

**Background:**

This study aimed to investigate whether schedule modification is safe and effective in patients intolerant to the standard eribulin dose and schedule.

**Methods:**

Patients with metastatic breast cancer (MBC) treated with both anthracycline and taxane and ≤ 3 prior regimens of chemotherapy for MBC received eribulin at the standard dose and schedule (1.4 mg/m^2^ on days 1 and 8 of a 21-day cycle) in the first cycle; change of dosing schedule (1.4 mg/m^2^ on days 1 and 15 of a 28-day cycle) was determined by change in neutrophil count, platelet count, aspartate aminotransferase, alanine aminotransferase, total bilirubin, serum creatinine, and non-hematological toxicity on day 8 of the first cycle or day 1 of the second cycle. Clinical benefit rate (CBR; primary endpoint), time to treatment failure (TTF), overall survival (OS), and safety were evaluated.

**Results:**

Of the 88 patients who were enrolled and received standard eribulin therapy in the first cycle, 42 patients were moved to the bi-weekly therapy group and 40 continued standard therapy. In the bi-weekly and standard therapy groups, mean relative dose intensity was 62.7 and 90.9%, CBR was 31.0 and 25.0%, median TTF was 81.5 and 75 days, and OS was 523 and 412 days, respectively. Neither group reported severe adverse events.

**Conclusion:**

This is the first study to show that a bi-weekly eribulin schedule is tolerable and has comparable efficacy in patients intolerant to the standard eribulin schedule.

**Clinical trial registration:**

University Hospital Medical Information Network (UMIN) Center (ID: UMIN 000008491).

**Electronic supplementary material:**

The online version of this article (10.1007/s12282-018-0843-y) contains supplementary material, which is available to authorized users.

## Introduction

Microtubule polymerization is a key process in cancer cell proliferation and a number of microtubule-targeting agents have been evaluated in preclinical and clinical studies [1]. Eribulin, an analog of halichondrin B, is a novel non-taxane microtubule dynamics inhibitor [2]. Because eribulin binds to a unique site on tubulin [3], it can be used to overcome taxane resistance and can be used in patients progressing after standard treatment with anthracycline- and taxane-containing regimens [4]. It has been approved in many countries for the treatment of metastatic breast cancer (MBC) in patients previously treated with chemotherapeutic agents like anthracycline- and taxane-based regimens [5–7]. In Japan, it is approved for the treatment of patients with inoperable or recurrent breast cancer [8] and is being investigated in patients who have not been previously treated with chemotherapy regimens for MBC [9].

Several clinical trials have demonstrated the antitumor activity and tolerability of eribulin. A global, multicenter, open-label, phase III, randomized study (EMBRACE study) demonstrated a significant and clinically meaningful improvement in overall survival (OS) with eribulin compared to that with treatment of physician’s choice in patients with heavily pre-treated MBC (median OS 13.1 vs. 10.6 months, *p* = 0.041) [10]. In another randomized phase III study (301 study), median OS in the eribulin group was longer than that in the capecitabine group, though the results were not significantly different [15.9 vs. 14.5 months, hazard ratio (HR) 0.88; 95% confidence interval (CI) 0.77–1.00; *p* = 0.056] [11]. A pooled analysis of these two phase III studies demonstrated that eribulin significantly improved OS compared to control in various subgroups of patients with pretreated MBC [12]. A single-arm, multicenter, open-label phase II study conducted in Japan, which enrolled MBC patients pretreated with an anthracycline and a taxane, has also shown both the efficacy and tolerability of eribulin as first- to fourth-line treatment [13].

Although eribulin demonstrated a manageable safety profile, the incidence of myelosuppression is high. In the EMBRACE trial, adverse events occurred in 98.8% patients receiving eribulin [10]. The incidence of both neutropenia and leukopenia was 98.8% in the Japanese phase II study [13]. In the MBC population known to have low quality of life (QOL) [14], adverse events may have further unfavorable impact on patient well-being and may lead to discontinuation of the eribulin therapy especially in severe cases. A comparison of the clinical benefits of eribulin versus capecitabine using health-related QoL (HRQoL) data from a phase III randomized trial in patients with MBC showed similar impact on patient functioning with no overall difference in HRQoL; however, patients who received eribulin showed worse systemic side-effects of chemotherapy such as dry mouth, different tastes, irritated eyes, feeling ill, hot flushes, headaches, and hair loss and lesser gastrointestinal toxicity compared to those receiving capecitabine [15]. The main aims of MBC treatment are prolonging OS and maintaining QOL. While non-hematologic toxicity is lower with eribulin than with other chemotherapeutic agents, discontinuation due to hematologic toxicity is a major problem of eribulin therapy. Management of neutropenia is important to continue eribulin treatment for clinical benefits. Since the standard dosing schedule of eribulin often leads to severe neutropenia, the management of eribulin dose and schedule is a key issue to decrease toxicity and continue treatment. Dose reduction of eribulin has been reported for patients with adverse events of higher grades in the previous studies [10, 13]. We conducted a phase II, non-randomized, prospective study to investigate whether schedule modification of bi-weekly eribulin therapy is safe and effective.

## Patients and methods

### Patients

Japanese women with MBC previously treated with an anthracycline and a taxane, and who had received up to three prior regimens of chemotherapy for MBC were included in the study. The main inclusion criteria were as follows: women aged ≥ 20 years, histologically confirmed human epidermal growth factor receptor 2 (HER2)-negative breast cancer, Eastern Cooperative Oncology Group performance status (ECOG PS) [16] of 0–2, measurable lesion in at least one dimension by computed tomography or magnetic resonance imaging based on Response Evaluation Criteria in Solid Tumors (RECIST) ver. 1.1. [17], neutrophil count ≥ 1000/μL, platelet count ≥ 100,000/μL, hemoglobin ≥ 9.0 g/dL, aspartate aminotransferase and alanine aminotransferase ≤ 3.0 times the upper limit of normal (ULN) or ≤ 5.0 × ULN in patients with hepatic metastases, total bilirubin ≤ 1.5 × ULN, serum creatinine ≤ 1.5 × ULN, and expected survival of ≥ 3 months.

Key exclusion criteria were as follows: systemic infection with fever ≥ 38.0 °C, pleural effusion, ascites or pericardial fluid requiring drainage, symptomatic brain metastasis, serious comorbidities (e.g., ischemic heart disease not controllable by treatment or heart disease such as arrhythmia, myocardial infarction < 6 months prior to study entry, complication of interstitial pneumonia or pulmonary fibrosis), second active cancer, inadequately controlled diabetes mellitus, pregnancy, breastfeeding, or women with childbearing potential.

### Study design

In the first cycle, 1.4 mg/m^2^ eribulin mesylate (equivalent to 1.23 mg/m^2^ of eribulin as free base) was administered intravenously over 2–5 min on days 1 and 8 of each 21-day cycle (the standard regimen). Based on the incidence of adverse events prior to eribulin administration on day 8 of the first cycle or day 1 of the second cycle, patients were allocated to standard therapy group or bi-weekly therapy group on day 1 of the second cycle. The criteria for dosing schedule modification included neutrophil count ≥ 1000/μL, platelet count ≥ 75,000/μL, aspartate aminotransferase and alanine aminotransferase ≤ 3.0 times the ULN or ≤ 5.0 × ULN in patients with hepatic metastases, total bilirubin ≤ 1.5 × ULN, serum creatinine ≤ 1.5 × ULN, non-hematological toxicity with grade ≤ 2. When patients met these criteria, they continued the standard regimen. But otherwise, they were allocated to the bi-weekly therapy, where 1.4 mg/m^2^ eribulin mesylate (equivalent to 1.23 mg/m^2^ of eribulin as free base) was administered on days 1 and 15 of each 28-day cycle. Eribulin was administered until disease progression, unacceptable toxicity, or withdrawal of consent. Table S1 shows the dosing schedule for patients switching to bi-weekly eribulin administration and requiring dose reduction and/or delayed administration.

After patients were allocated to the two groups, they continued to receive eribulin treatment in the same group. Dose reduction to 1.1 mg/m^2^ was permitted to manage toxicity of grade ≥ 3 febrile neutropenia, grade ≥ 3 neutropenia with infection requiring antibiotic treatment, grade 4 thrombocytopenia, or grade ≥ 3 non-hematological toxicity.

Eribulin administration was discontinued for patients requiring dose reduction to < 1.1 mg/m^2^ or delay in administration > 2 weeks. Administration of granulocyte colony-stimulating factor (G-CSF) was permitted in the event of grade 4 neutropenia or grade ≥ 3 febrile neutropenia; however, the use of G-CSF for preventing neutropenia was not permitted.

### Rationale for dosing schedule modification

Table S2 shows the theoretical dose intensity of eribulin in the standard schedule and bi-weekly schedule. When patients were unable to continue eribulin treatment on the standard regimen (1.4 mg/m^2^ on days 1 and 8 of each 21-day cycle), the dose reduction to 1.1 mg/m^2^ on the same dosing schedule had been determined in the previous studies [10, 11] in which the dose intensity decreased to 0.73 mg/m^2^ per week. A similar (0.70 mg/m^2^) dose intensity was maintained in bi-weekly schedule with the dose of 1.4 mg/m^2^ in this study.

### Assessments

The primary endpoint of this study was clinical benefit rate (CBR, defined as the proportion of patients who achieved complete response, partial response, or stable disease for ≥ 6 months) based on RECIST v. 1.1. Secondary endpoints included time to treatment failure (TTF), OS, and safety. TTF was defined as the time from initiation of eribulin to treatment discontinuation for any reason, including disease progression, treatment toxicity, patient preference, or death caused by treatment. OS was calculated as the time from the initiation of eribulin until death from any cause. For safety, adverse events were recorded and graded according to the National Cancer Institute Common Terminology Criteria for Adverse Events (version 4.0, Japanese version) [18], and were coded according to Medical Dictionary for Regulatory Activities, Japanese version [19]. In addition, ad hoc analyses were performed: factors influencing the schedule modification of eribulin to bi-weekly administration were evaluated, and subgroup analyses were performed in elderly (aged ≥ 65 years) and younger (aged < 65 years) patients.

The study protocol was approved by local institutional review boards and ethics committees. This study was conducted in accordance with the Japanese Guidelines for Clinical Research of the Ministry of Health, Labour and Welfare and the Declaration of Helsinki, as well as other applicable regulatory requirements. All participants provided written informed consent prior to the study entry. The present study has been registered with the University Hospital Medical Information Network (UMIN) Center (ID: UMIN 000008491) [20].

### Statistical methods

The sample size was calculated to evaluate the null hypothesis that the true CBR was 15% and the alternative hypothesis that the CBR was ≥ 30%, with a type I error level of 0.1 and type II error level of 0.20. The minimum target sample size was set at *N* = 70 and we aimed to recruit 80 patients with the consideration that ~ 10% of the recruited patients would not be evaluable. In the phase II clinical trial of eribulin in Japan, patients had a CBR of 27.5% with a median of 3 prior chemotherapy sessions. In addition, 33.3% of the patients could not be administered eribulin on day 8 of cycle 1 because of myelosuppression. Based on these findings, we assumed that 50% of the patients would not be eligible for eribulin on day 8 of cycle 1 in clinical practice.

The data cut-off date was February 25, 2016. The efficacy data were assessed in the eligible population who received at least one dose of the study drug and had evaluable efficacy data (full analysis set; FAS). The CBR and two-sided 95% CIs were calculated based on binominal distribution. TTF and OS (estimated median with 95% CI) were calculated using Kaplan–Meier methods. The safety data were assessed in the eligible population who received at least one dose of the study drug (safety population), which were presented by descriptive analyses. SAS software (version 9.4) (SAS Institute Inc., Cary, NC, USA) was used for all analyses.

## Results

**Fig. 1:**
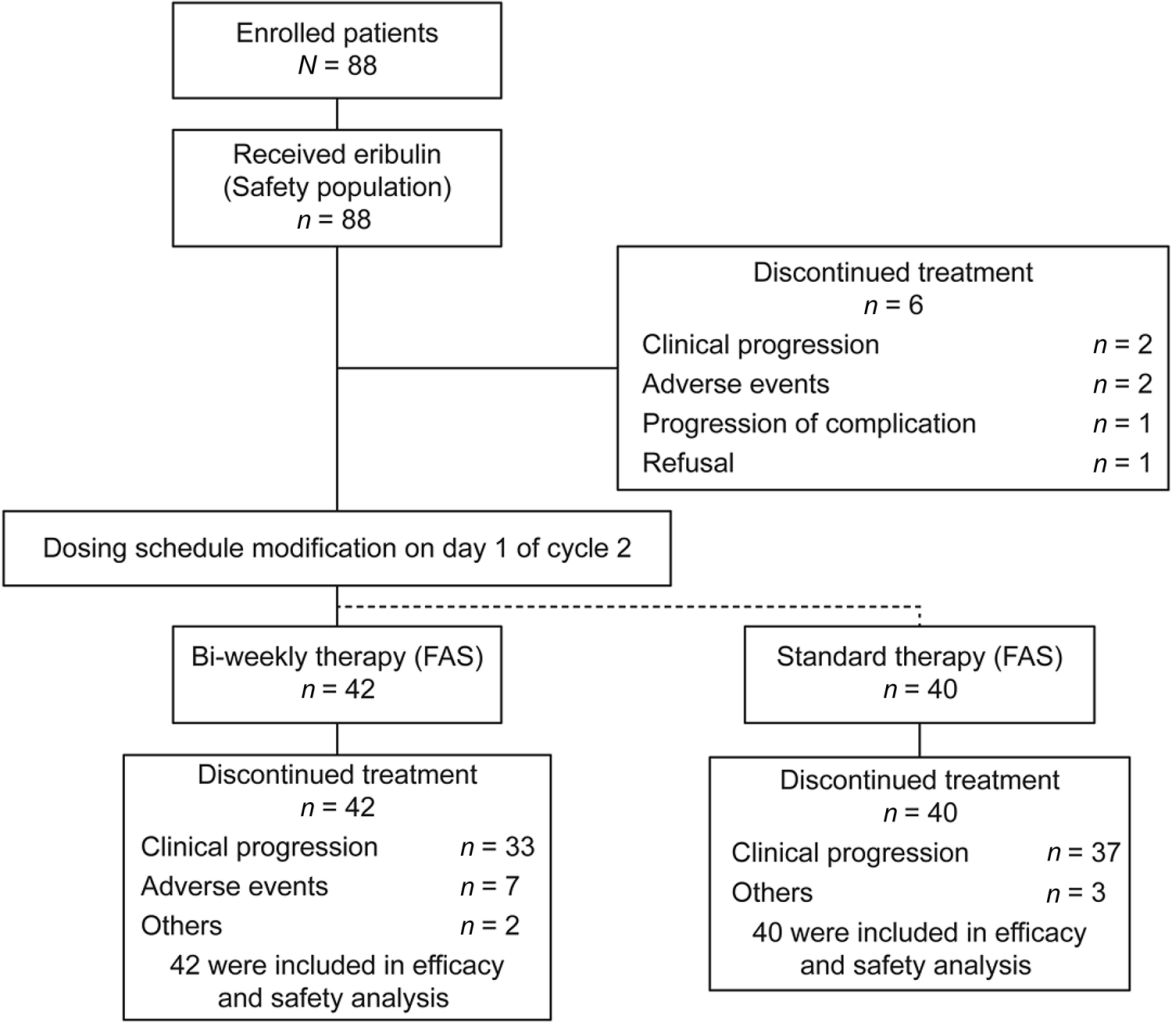
Patient disposition. *FAS* full analysis set

We conducted a phase II, prospective, non-randomized, open-label, multicenter study at 16 sites in Japan. A total of 88 patients were recruited in the study between July 2012 and April 2014. All 88 patients received at least one dose of eribulin. A total of 82 patients were eligible for efficacy analyses (FAS); 40 patients continued the standard eribulin therapy (standard group) and the remaining 42 patients required schedule modification (bi-weekly group) (Fig. 1).

The baseline characteristics of patients are summarized in Table 1. Although patients in bi-weekly group were intolerant to standard regimens, the characteristics and medical history were similar between the two groups.

**Table 1:** Baseline characteristics of patients

	Overall	Standard	Bi-weekly
	*N* = 88	*N* = 40	*N* = 42
Median age, (range) years	59.5 (37–80)	61.0 (37–80)	60.0 (40–77)
ECOG performance status, *n* (%)			
0	59 (67.0)	26 (65.0)	30 (71.4)
1	28 (31.8)	14 (35.0)	12 (28.6)
2	1 (1.1)	0	0
ER/PgR positive, *n* (%)	54 (61.4)	23 (57.5)	27 (64.3)
Triple-negative, *n* (%)	34 (38.6)	17 (42.5)	15 (35.7)
No. of chemotherapies for MBC, *n* (%)			
0	15 (17.0)	5 (12.5)	9 (21.4)
1	23 (26.1)	14 (35.0)	8 (19.0)
2	27 (30.7)	10 (25.0)	14 (33.3)
3	23 (26.1)	11 (27.5)	11 (26.2)
No. of prior chemotherapy regimens (median)	2	2	2
Prior chemotherapy for MBC^a^, *n* (%)			
FEC	26 (29.5)	10 (25.0)	13 (31.0)
Paclitaxel + bevacizumab	26 (29.5)	13 (32.5)	10 (23.8)
nab-Paclitaxel	12 (13.6)	3 (7.5)	8 (19.0)
Paclitaxel	13 (14.8)	7 (17.5)	6 (14.3)
S-1	12 (13.6)	4 (10.0)	6 (14.3)
XC	11 (12.5)	5 (12.5)	6 (14.3)
Capecitabine	15 (17.0)	9 (22.5)	5 (11.9)
Docetaxel	6 (6.8)	4 (10.0)	2 (4.8)
Prior surgery, *n* (%)	79 (89.8)	36 (90.0)	37 (88.1)
Prior radiation therapy, *n* (%)	29 (33.0)	10 (25.0)	15 (35.7)
Most common metastatic sites^a^, *n* (%)			
Liver	47 (53.4)	21 (52.5)	24 (57.1)
Bone	41 (46.6)	22 (55.0)	18 (42.9)
Lymph nodes	24 (27.3)	10 (25.0)	12 (28.6)
Lung	19 (21.6)	9 (22.5)	7 (16.7)
Axilla	14 (15.9)	6 (15.0)	7 (16.7)
No. of metastatic sites, median	1	1	2

Overall population = safety population = 88 patients

6 patients were excluded (2 due to progressive disease, 2 due to adverse events, 1 due to complication, 1 due to refusal)

Standard therapy group = efficacy analysis population = 40 patients

Biweekly therapy group = efficacy analysis population = 42 patients

*ECOG* Eastern Cooperative Oncology Group, *ER* estrogen receptor, *PgR* progesterone receptor, *FEC* 5-FU + epirubicin + cyclophosphamide, *MBC* metastatic breast cancer, *S1* tegafur/gimeracil/oteracil potassium, *XC* capecitabine + cyclophosphamide

^a^Data shown are for > 10% of patients

Eribulin was administered for a median of 73.0 days (range 14–365) in the overall population, 77.0 days (range 35–322) in the standard group, and 87.0 days (range 28–365) in the bi-weekly group. Mean relative dose intensity was 76.9% in the overall population, 90.9% in the standard group, and 62.7% in the bi-weekly group when the dose intensity of 0.93 mg/m^2^/week was considered as 100%.

### Efficacy outcomes

The ORR and CBR were 19.3% (95% CI 11.7–29.1) and 26.1% (95% CI 17.3–36.6) in the overall population, 20.0% (95% CI 9.1–35.6) and 25.0% (95% CI 12.7–41.2) in the standard group, and 21.4% (95% CI 10.3–36.8) and 31.0% (95% CI 17.6–47.1) in the bi-weekly group, respectively (Table 2).

**Table 2:** Response rate

	Overall	Standard	Bi-weekly
	*N* = 88	*N* = 40	*N* = 42
Best overall response, *n* (%)			
Complete response	2 (2.3)	1 (2.5)	1 (2.4)
Partial response	15 (17.0)	7 (17.5)	8 (19.0)
Stable disease	23 (26.1)	9 (22.5)	13 (31.0)
Stable disease ≥ 6 months	6 (6.8)	2 (5.0)	4 (9.5)
Progressive disease	37 (42.0)	21 (52.5)	15 (35.7)
Not evaluable	5 (5.7)	0	1 (2.4)
Objective response rate, *n* (%)	17 (19.3)	8 (20.0)	9 (21.4)
95% CI	11.7–29.1	9.1–35.6	10.3–36.8
Clinical benefit rate, *n* (%)	23 (26.1)	10 (25.0)	13 (31.0)
95% CI	17.3–36.6	12.7–41.2	17.6–47.1
Time to treatment failure, days	77	75	81.5
95% CI	70–95	69–119	73–107
Overall survival, days	427	412	523
95% CI	300–701	275–713	350–828

*CI* confidence interval

The median TTF and OS were 77 days (95% CI 70–95) and 427 days (95% CI 300–701) in the overall population, 75 days (95% CI 69–119) and 412 days (95% CI 275–713) in the standard group, and 81.5 days (95% CI 73–107) and 523 days (95% CI 350–828) in the bi-weekly group (Table 2).

### Safety outcomes

In the overall population, the most common adverse events with eribulin were leukopenia (83.0%), neutropenia (77.3%), anemia (58.0%), fatigue (56.8%), and alopecia (56.8%) (Table 3). The most common grade 3 and 4 AEs were neutropenia (59.1%) and leukopenia (45.5%). In the bi-weekly group, the incidence rates of leukopenia and neutropenia of any grade were 95.2 and 92.9%, respectively, whereas in the standard group, the corresponding incidence rates were 72.5 and 62.5%, respectively. Febrile neutropenia was observed in one (2.5%) patient in the standard group and five (11.9%) patients in the bi-weekly group (Table 3).

**Table 3:** Adverse events

	Overall (*N* = 88)			Standard (*N* = 40)			Bi-weekly (*N* = 42)		
	Any grade	Grade 3	Grade 4	Any grade	Grade 3	Grade 4	Any grade	Grade 3	Grade 4
Hematologic events, *n* (%)									
Leukopenia	73 (83.0)	32 (36.4)	8 (9.1)	29 (72.5)	9 (22.5)	2 (5.0)	40 (95.2)	22 (52.4)	3 (7.1)
Neutropenia	68 (77.3)	28 (31.8)	24 (27.3)	25 (62.5)	9 (22.5)	3 (7.5)	39 (92.9)	18 (42.9)	19 (45.2)
Anemia	51 (58.0)	3 (3.4)	0	24 (60.0)	3 (7.5)	0	23 (54.8)	0	0
Thrombocytopenia	16 (18.2)	3 (3.4)	0	9 (22.5)	2 (5.0)	0	5 (11.9)	0	0
Febrile neutropenia	7 (8.0)	7 (7.0)	0	1 (2.5)	1 (2.5)	0	5 (11.9)	5 (11.9)	0
Non-hematologic events, *n* (%)									
AST increased	45 (51.1)	3 (3.4)	0	18 (45.0)	0	0	24 (57.1)	2 (4.8)	0
Alopecia	50 (56.8)	0	0	26 (65.0)	0	0	23 (54.8)	0	0
Peripheral sensory neuropathy	42 (47.7)	1 (1.1)	0	18 (45.0)	0	0	22 (52.4)	1 (2.4)	0
Fatigue	50 (56.8)	5 (5.7)	0	24 (60.0)	4 (10.0)	0	22 (52.4)	1 (2.4)	0
Malaise	46 (52.3)	0	0	22 (55.0)	0	0	19 (45.2)	0	0
ALT increased	37 (42.0)	2 (2.3)	0	17 (42.5)	0	0	18 (42.9)	2 (4.8)	0
Nausea	32 (36.4)	1 (1.1)	0	15 (37.5)	0	0	17 (40.5)	1 (2.4)	0
Dysgeusia	32 (36.4)	0	0	18 (45.0)	0	0	12 (28.6)	0	0
Mucositis oral	21 (23.9)	1 (1.1)	0	9 (22.5)	0	0	11 (26.2)	1 (2.4)	0
Pain	24 (27.3)	3 (3.4)	0	12 (30.0)	0	0	9 (21.4)	2 (4.8)	0
Constipation	18 (20.5)	0	0	8 (20.0)	0	0	8 (19.0)	0	0
Peripheral motor neuropathy	14 (15.9)	2 (2.3)	0	4 (10.0)	0	0	8 (19.0)	1 (2.4)	0
Nail discoloration	11 (12.5)	0	0	5 (12.5)	0	0	6 (14.3)	0	0
Edema limbs	9 (10.2)	0	0	4 (10.0)	0	0	5 (11.9)	0	0
Rash maculo-papular	4 (4.5)	1 (1.1)	0	0	0	0	4 (9.5)	1 (2.4)	0
Vomiting	5 (5.7)	0	0	2 (5.0)	0	0	3 (7.1)	0	0
Pharyngitis	4 (4.5)	0	0	1 (2.5)	0	0	3 (7.1)	0	0
Diarrhea	5 (5.7)	0	0	3 (7.5)	0	0	2 (4.8)	0	0
Blood bilirubin increased	7 (8.0)	0	0	3 (7.5)	0	0	2 (4.8)	0	0
Nail loss	1 (1.1)	0	0	0	0	0	1 (2.4)	0	0
Skin hyperpigmentation	3 (3.4)	0	0	2 (5.0)	0	0	1 (2.4)	0	0
Creatinine increased	9 (10.2)	2 (2.3)	0	7 (17.5)	2 (5.0)	0	1 (2.4)	0	0

*ALT* alanine aminotransferase, *AST* aspartate aminotransferase

**Fig. 2:**
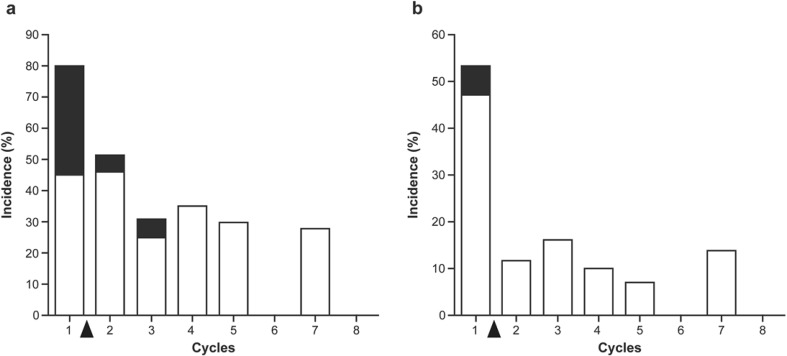
Frequency of grade 3/4 neutropenia and leukopenia during treatment cycles in bi-weekly group. **a** Neutropenia; **b** leukopenia. White and black columns represent grades 3 and 4, respectively. Black arrow indicates the timing of the schedule modification based on the adverse events in the first cycle

Among patients in the bi-weekly group, grade 3/4 neutropenia was the major adverse event leading to schedule modification and reported in 34 patients (81.0%) (Table 4). Grade 3/4 leukopenia was reported in 22 patients (52.4%). After transition to the bi-weekly schedule, grade 3/4 neutropenia reduced to 61.9% and grade 3/4 leukopenia to 26.2%. The frequencies of grade 3/4 neutropenia and leukopenia during treatment cycles are shown in Fig. 2. By the modification of dosing schedule, the frequencies of these toxicities were dramatically decreased and the low incidences were maintained thereafter. Only 2 (4.8%) patients experienced febrile neutropenia after schedule modification. The adverse events leading to discontinuation of eribulin were neutropenia (6 patients; 14.3%) and peripheral sensory neuropathy (1 patient; 2.4%) in the bi-weekly group (data not shown). No patients discontinued the study due to adverse event(s) in the standard group.

**Table 4:** Adverse events: before/after schedule modification (bi-weekly group; *N* = 42)

Adverse events, *n* (%)	Before schedule modification			After schedule modification		
	Any grade	Grade 3	Grade 4	Any grade	Grade 3	Grade 4
Leukopenia	39 (92.9)	19 (45.2)	3 (7.1)	34 (81.0)	11 (26.2)	0
Neutropenia	39 (92.9)	19 (45.2)	15 (35.7)	35 (83.3)	18 (42.9)	8 (19.0)
Anemia	21 (50.0)	0	0	22 (52.4)	0	0
AST increased	21 (50.0)	1 (2.4)	0	22 (52.4)	2 (4.8)	0
Hypoalbuminemia	21 (50.0)	0	0	21 (50.0)	0	0
Fatigue	20 (47.6)	0	0	20 (47.6)	1 (2.4)	0
Peripheral sensory neuropathy	19 (45.2)	0	0	22 (52.4)	1 (2.4)	0
Alopecia	18 (42.9)	0	0	23 (54.8)	0	0
Malaise	17 (40.5)	0	0	19 (45.2)	0	0
ALT increased	15 (35.7)	1 (2.4)	0	12 (28.6)	2 (4.8)	0
Nausea	12 (28.6)	0	0	13 (31.0)	1 (2.4)	0
Dysgeusia	10 (23.8)	0	0	9 (21.4)	0	0
Peripheral motor neuropathy	8 (19.0)	0	0	8 (19.0)	1 (2.4)	0
Pain	7 (16.7)	1 (2.4)	0	9 (21.4)	2 (4.8)	0
Mucositis oral	6 (14.3)	1 (2.4)	0	11 (26.2)	0	0
Constipation	4 (9.5)	0	0	7 (16.7)	0	0
Thrombocytopenia	4 (9.5)	0	0	2 (4.8)	0	0
Febrile neutropenia	3 (7.1)	3 (7.1)	0	2 (4.8)	2 (4.8)	0
Blood bilirubin increased	0	0	0	2 (4.8)	0	0
Creatinine increased	0	0	0	1 (2.4)	0	0

*ALT* alanine aminotransferase, *AST* aspartate aminotransferase

### Ad hoc analyses

In the ad hoc analyses, we determined the association of dosing schedule modification with the baseline characteristics including age, neutrophil count (Table 5), presence of liver metastases, aspartate aminotransferase level, alanine aminotransferase level, and blood albumin level (data not shown). However, these were not significantly associated with the schedule modification of eribulin (Table 5).

**Table 5:** Subgroup analyses (CBR)

	*N*	Overall (*N* = 88)	*N*	Standard (*N* = 40)	*N*	Bi-weekly (*N* = 42)
All	88	23 (26.1)	40	10 (25.0)	42	13 (31.0)
Hormone receptor status (ER and/or PgR)						
Positive	54	16 (29.6)	23	7 (30.4)	27	9 (33.3)
Negative	34	7 (20.6)	17	3 (17.6)	15	4 (26.7)
No. of chemotherapy regimens for advanced or MBC						
0	15	7 (46.7)	5	2 (40.0)	9	5 (55.6)
1	23	4(17.4)	14	2 (14.3)	8	2(25.0)
2	27	7 (25.9)	10	4 (40.0)	14	3 (21.4)
3	23	5(21.7)	11	2 (18.2)	11	3 (27.3)
Relative dose intensity						
> 75%	50	10 (20.0)	36	9 (25.0)	8	1 (12.5)
< 75%	38	13 (34.2)	4	1 (25.0)	34	12 (35.3)
Neutropenia (cycle 1, day 8)						
> 1000	57	15 (26.3)	39	10 (25.6)	14	5 (35.7)
< 1000	30	8 (26.7)	0	0 (0.0)	28	8 (28.6)
Age						
≥ 65	26	9 (34.6)	14	5 (35.7)	12	4 (33.3)
< 65	56	15 (26.8)	26	5 (19.2)	30	9 (30.0)

*CBR* clinical benefit rate, *ER* estrogen receptor, *PgR* progesterone receptor, *MBC* metastatic breast cancer

In elderly patients (aged ≥ 65 years), the CBR (Table 5) and ORR (data not shown) were 35.7 and 21.4% in the standard group, and 33.3 and 25.0% in the bi-weekly group, respectively. On the other hand, in younger patients (aged < 65 years), the CBR and ORR were 19.2 and 19.2% in the standard group, and 30.0 and 20.0% in the bi-weekly group, respectively. Most likely, elderly patients may be susceptible to myelosuppression and be unable to continue on the standard schedule of eribulin. Bi-weekly eribulin therapy is an effective and safe option for elderly patients.

## Discussion

Management of eribulin dose and schedule is a key issue to decrease toxicity and increase efficacy. Among patients who required schedule modification, the majority of patients (76.2%) needed it due to neutropenia. Although leukopenia and neutropenia were more frequently observed in patients who required schedule modification compared with those who continued the standard regimen of eribulin, before and after switching to bi-weekly administration, incidence of grade 3/4 leukopenia decreased from 52.4 to 26.2% and grade 3/4 neutropenia decreased from 81.0 to 61.9%. By modification of dosing schedule, the frequencies of these toxicities were dramatically decreased in the second cycle and thereafter. There was no incidence of grade 4 of leukopenia and neutropenia after 4 cycles in bi-weekly therapy, suggesting that the majority of patients transferred to the bi-weekly regimen could continue eribulin therapy safely for 8 courses. Furthermore, the frequency of hospital visits was 20% lower with the bi-weekly schedule as compared with the standard schedule.

The efficacy results of patients who required schedule modification were comparable to those of patients who continued the standard eribulin regimen. In addition, our ad hoc analyses demonstrated that the tolerability of eribulin was comparable between patients aged < 65 years and those aged ≥ 65 years. Thus, eribulin may also be beneficial for elderly patients.

The efficacy results with the modified bi-weekly schedule in the present study were comparable to those of a previous phase II study conducted in Japan with the standard regimen [13]. CBR and ORR were 31.0 and 21.4% in patients who received bi-weekly therapy of eribulin, compared to 27.5 and 21.3%, in patients on the standard regimen in the previous phase II study [13]. The median OS was also similar and was 523 days (17.2 months) in the present study and 11.1 months in the previous phase II study [13]. In addition, the adverse events observed in the present study were consistent with those in previous phase II/III studies conducted globally or in Japan [10–13, 21–25], and no new safety signals were detected. Hematological toxicities are common with eribulin. In the present study, the majority of patients experienced hematologic adverse events, including neutropenia and leucopenia. In the overall population, grade 3/4 neutropenia was observed in 59.1%, and grade 3/4 leukopenia was observed in 45.5% of patients, figures which are relatively low compared to the previous phase II study conducted in Japan (95.1 and 74.1%, respectively) [13]. Moreover, the incidence of grade 3/4 febrile neutropenia in the present study was 8.0%, which was lower than the 13.6% in the previous phase II study [13]. Only 2 (4.8%) patients experienced febrile neutropenia after schedule modification.

To our knowledge, this is the first study investigating safety and effectiveness of bi-weekly administration of eribulin in patients with MBC; however, this study has some limitations. This was a non-randomized study: the patients were not randomly assigned to the standard group and bi-weekly group and the patients in the bi-weekly group were those who were unable to continue the standard regimen of eribulin. Moreover, sample sizes of both groups (standard schedule and bi-weekly schedule) as well as subgroups were small. Therefore, the findings should be interpreted with caution.

In conclusion, this phase II study involving patients with MBC demonstrated that bi-weekly administration of eribulin in patients who were intolerant to the standard regimen of eribulin had antitumor activity comparable to the standard therapy. Since the risk of adverse events was reduced after switching to bi-weekly regimen in the patients who were not tolerant to the standard regimen of eribulin, bi-weekly administration of eribulin might be an alternative option for such patients. In addition, the bi-weekly schedule may contribute to the future exploration of combination therapy of eribulin with other cancer agents.

## Electronic supplementary material

Below is the link to the electronic supplementary material.

Supplementary material 1 (DOCX 36 kb)
