# Supplementary material for: Bi-weekly eribulin therapy for metastatic breast cancer: a multicenter phase II prospective study (JUST-STUDY)
Source: Breast Cancer. 2018 Feb 12;25(4):438–46. doi: 10.1007/s12282-018-0843-y (PMC5996009; doi:10.1007/s12282-018-0843-y)
Supplement: Supplementary file 1 — Supplementary material 1 (DOCX 36 kb) [file 12282_2018_843_MOESM1_ESM.docx]

**Supplementary Material**

**Table S1** Dosing schedule for patients switching to bi-weekly eribulin administration, and requiring dose reduction and/or delayed administration

| **Day** | **1** | **8** | **15** | **22** | **29** | **36** | **43** |
| --- | --- | --- | --- | --- | --- | --- | --- |
| **Standard schedule** | | | | | | | |
| 1.4 mg/m^2^, standard | X | X |  | X | X |  | X |
| **Bi-weekly schedule** | | | | | | | |
| 1.4 mg/m^2^, standard | X | NR |  |  |  |  |  |
| 1.4 mg/m^2^, bi-weekly |  |  | X |  | X |  | X |
| **Bi-weekly schedule when no recovery within 1 week from day 8 of cycle 1 (one more week of cessation of administration followed by restarting the administration)** | | | | | | | |
| 1.4 mg/m^2^, standard | X | NR |  |  |  |  |  |
| 1.4 mg/m^2^, bi-weekly |  |  | NR | X |  | X |  |
| **Bi-weekly schedule when no recovery by day 1 of cycle 2 (one more week of cessation of administration followed by restarting the administration)** | | | | | | | |
| 1.4 mg/m^2^, standard | X | X |  | NR |  |  |  |
| 1.4 mg/m^2^, bi-weekly |  |  |  |  | X |  | X |
| **Bi-weekly schedule when no recovery within 1 week from day 1 of cycle 2 (one more week of cessation of administration followed by restarting the administration)** | | | | | | | |
| 1.4 mg/m^2^, standard | X | X |  | NR |  |  |  |
| 1.4 mg/m^2^, bi-weekly |  |  |  |  | NR | X |  |

NR, no recovery

**Table S2** Theoretical dose intensity of eribulin

|  | Eribulin Standard Schedule | Bi-weekly Schedule |
| --- | --- | --- |
|  | iv on days 1 and 8 every 3 weeks | iv on days 1 and 15 every 4 weeks |
| Eribulin 1.4 mg/m^2^ | 0.93 | 0.70 |
| Eribulin 1.1 mg/m^2^ | 0.73 | 0.55 |

Dose intensity was shown as the total dose per week (mg/m^2^). iv, intravenously.
